# Kinetics of Apoptosis and Expression of Apoptosis-Related Proteins in Rat CA3 Hippocampus Cells After Experimental Diffuse Brain Injury

**DOI:** 10.1007/s12013-013-9597-5

**Published:** 2013-04-05

**Authors:** Jianliang Chen, Xuesong Li, Jiandong Qiu, Hengxing You, Qingping Zhang, Guangyu Dong, You Zuo

**Affiliations:** 1 Department of Neurosurgery, The Fourth People’s Hospital of Shenzhen (Futian People’s Hospital), Shenzhen, 518023 China; 2 Department of Neurosurgery, Huizhou Municipal Central Hospital, Huizhou, 516001 China; 3 Department of Neurourgery, The Longgang People’s Hospital, Shenzhen, 518023 China; 4 Department of Neurourgery, The Yantian People’s Hospital, Shenzhen, 518023 China

**Keywords:** Diffuse brain injury, Apoptosis, Gene expression, Rats, Hippocampus

## Abstract

The present study examined kinetics of apoptosis and expression of apoptosis-related proteins Bcl-2, Bax, and caspase-3 in the CA3 hippocampus cells after diffuse brain injury (DBI) induced experimentally in rats. Percentage of apoptotic cells and expressions of above proteins were examined by flow cytometry and immunohistochemistry. Substantial neuronal apoptosis was documented in the CA3 hippocampus cells after DBI (22.26 ± 2.97 % at 72 h after DBI vs. 2.92 ± 0.88 % in sham-operated animals). Expression of Bc1-2 decreased, while expression of Bax and caspase-3 increased after DBI, with caspase-3 expression peaking after that of Bax (72 vs. 48 h, respectively). Further, the Bc1-2/Bax expression ratio decreased prior to increase of caspase-3 expression. In conclusion, cell apoptosis and altered expressions of Bcl-2, Bax, and caspase-3 are present in the CA3 region of hippocampus after experimental DBI. Changes in the Bc1-2/Bax expression ratio may facilitate activation of caspase-3 and aggravate neuronal apoptosis after brain injury.

## Introduction

Apoptosis is a programmed cell death occurring under physiological and pathological conditions. Cell apoptosis occurs in patients after traumatic brain injury (TBI) [1], also during the secondary damage after TBI [2]. While proteins Bcl-2, Bax, and caspase-3 were shown to regulate apoptosis induced by TBI [3], their expressions and relations to apoptosis after the closed head diffuse brain injury (DBI) have rarely been reported. Here, we examined the development of apoptosis in CA3 hippocampus, and expression of Bcl-2, Bax, and caspase-3 in adult rats after the closed head DBI. The obtained results identify potential therapeutic targets for anti-apoptotic and neuroprotective therapy after the closed head DBI.

## Materials and Methods

### Experimental DBI

The studies presented here were approved by the institutional Animal Ethics committee and conformed to the national and international ethical standards and regulations on research involving animals. Adult male Sprague–Dawley rats [*n* = 70, mean (± SD) weight of 375 ± 15 g] were randomly divided into the sham-operated and DBI groups. Each of these groups was further subdivided into subgroups, each comprising 10 animals. The subgroups represented the studied time points (i.e., 0, 6, 12, 24, 48, 72 h and 7 days) after experimental injury. Five animals in each subgroup were used for immunohistochemistry, while the other five were used for the flow cytometry studies of apoptosis. Experimental DBI was induced as per previously published method [2]. Specifically, anesthetized rats were injured using a weight-drop device [2] which had a mass-height combination of 400 g × 120 cm. The rats in the sham-operated group were exposed to the same procedures as the animals in the trauma group with the exception of the exposure to the weight-drop injury.

### Flow Cytometry Analysis of Cell Apoptosis in CA3 Hippocampus

At 6, 12, 24, 48, 72 h and 7 days after DBI, the animals were anesthetized with intraperitoneal injection of 10 % chloral hydrate (2 ml/kg) and perfused transcardially through aorta with 250–300 ml of ice-cold heparinized saline at a flow rate of 80–100 ml/min. After decapitation, brains were quickly removed, and coronal sections (5 μm) of brain tissue were prepared starting 3.5 mm behind the anterior fontanelle. The sections were flash-frozen in liquid nitrogen and kept at −80 °C. Brains were further dissected, and 2 mg of brain tissue from the right side CA3 hippocampus were collected and resuspended in phosphate-buffered saline (PBS; pH 7.4). The obtained cell suspension was filtered through 200 μm mesh nylon net and centrifuged at 1,500×*g* for 5 min. The supernatant was discarded, and cells were fixed in ice-cold 70 % ethanol and stored at −20 °C. After storage, cells were re-centrifuged (1,500×*g*, 5 min) and ethanol was discarded. Cells were resuspended in PBS, and cell concentration was adjusted to 1 × 10^6^/l. Samples were stained with annexin V/propidium iodide (PI) (Becton–Dickinson, Franklin, USA), with controls set for each sample. Fluorescence of 10,000 cells was analyzed using the Epics Altra flow cytometer (Beckman Coulter, Franklin, USA). The cells stained with either annexin V or PI emit, respectively, green or red fluorescence, while the annexin V and PI negative cells are not fluorescent. The cells were classified as live cells (annexin V negative/PI negative), apoptotic cells (annexin V positive/PI negative), necrotic cells (annexin V positive/PI positive), and mechanically injured cells (annexin V negative/PI positive).

### Immunohistochemistry Assay for Expression of Bcl-2, Bax, and Caspase-3 in CA3 Hippocampus

Rats were anesthetized as described above and perfused transcardially with 300 ml of ice-cold heparinized saline with a flow rate of 80–100 ml/min, followed by 300 ml of 4 % paraformaldehyde (pH 7.4) at a flow rate of 20–40 ml/min. After decapitation, brains were removed and further immersion-fixed for 30 min in 4 % paraformaldehyde. Brain tissue was coronally sectioned in 6 μm sections starting 3.5 mm behind the anterior fontanelle, immersed in 4 % paraformaldehyde, and stored at 4 °C. Then, each sample was cut into six sections of 6 μm each, dehydrated and mounted. Out of each six samples, three were used to analyze expression of Bcl-2, Bax, and caspase-3, while the remaining three samples served as negative controls.

Samples were stained with rabbit polyclonal antibodies against rat Bcl-2, Bax, or caspase-3, followed by staining with the avidin–biotin–peroxidase staining kit (Wuhan Boster Bio-Engineering Ltd., Wuhan, China). Samples were then evaluated under microscope. Cells stained positive for Bcl-2 exhibited brown color staining inside the cytoplasm or nuclear membrane, while negatively stained did not develop any color. The percentage of positively stained cells was counted per every 100 cells from the right side CA3 hippocampus under magnification of 400. The magnitude of protein expression was ranked as follows: negative staining = zero, specimens with fewer than 5 % positive cells = 1, 6–15 % of positive cells = 2, 16–30 % positive cells = 3, 31–50 % = 4, and specimens with >50 % positive cells = 5.

### Statistical Analysis

Ranked expressions of Bcl-2, Bax, and caspase-3, and the number of apoptotic cells are presented as mean ± SD. The differences were analyzed using the ANOVA or *t* test (SPSS10.0, IBM, New York, USA). The *p* value of <0.05 was considered statistically significant.

## Results

### Apoptosis in the CA3 Region of Hippocampus in Experimental DBI

**Fig. 1:**
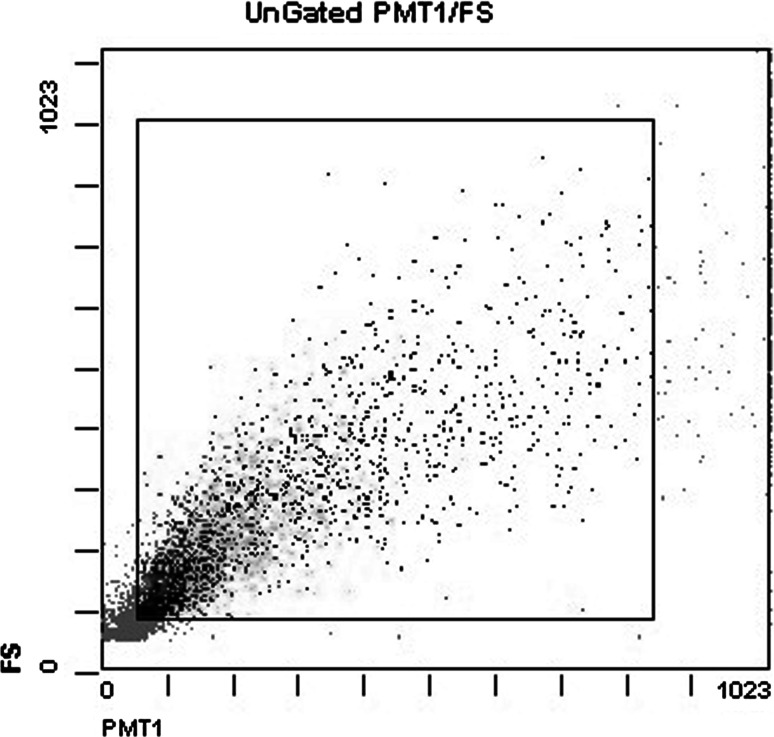
Light scattering properties of the rat CA3 hippocampus cells. The *x* and *y* axes represent, respectively, the side and forward light scattering properties of rat hippocampus cells

The flow cytometry analysis revealed that cells could not be separated into subpopulations based on forward and side light scatter characteristics (Fig. 1). Therefore, the results below represent apoptosis rates in the mixed cell population.

**Fig. 2:**
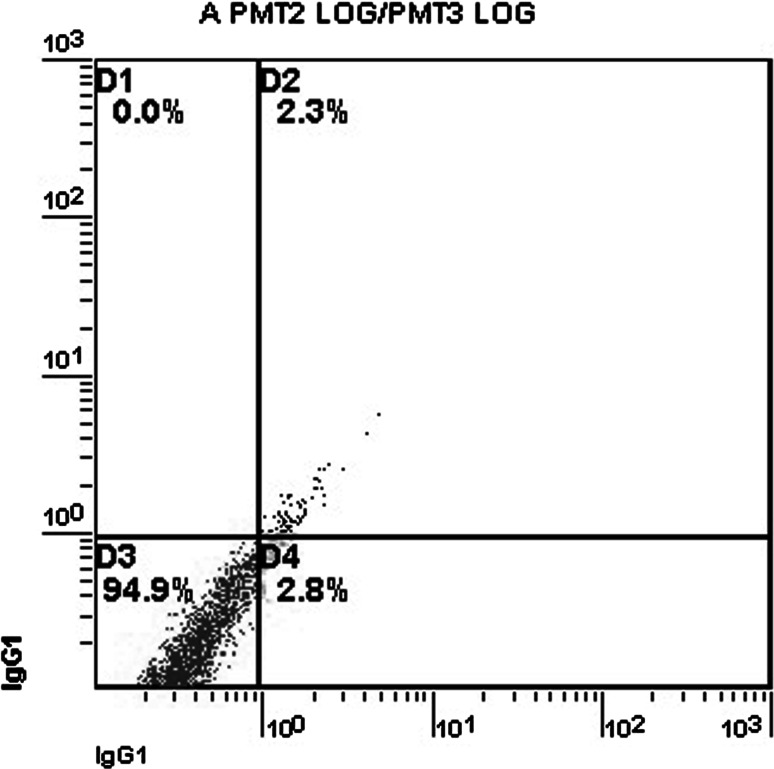
Lack of apoptosis in rat CA3 hippocampus cells in sham-operated animals. The cells are divided into four different populations based on apoptosis/necrosis staining. The cell populations are presented as percentage of total cells. The *x* and *y* axes represent cell numbers. Quadrant D1 represents necrotic cells, quadrant D2 late apoptotic cells, quadrant D3 intact cells, and quadrant D4 early apoptotic cells

**Fig. 3:**
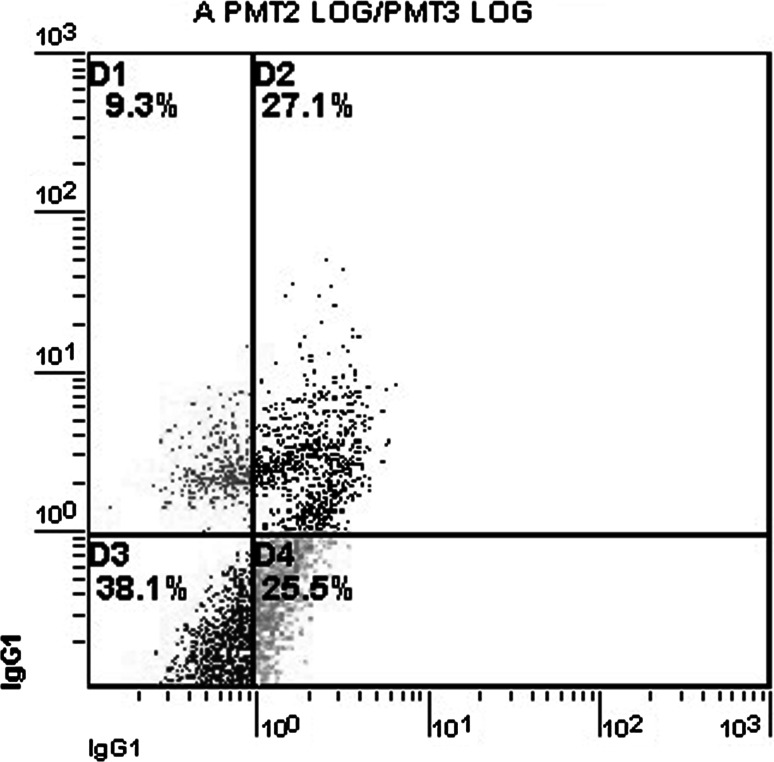
Substantial apoptosis in rat CA3 hippocampus cells 72 h after brain injury. The cells are divided into four different populations based on apoptosis/necrosis staining. The cell populations are presented as percentage of total cells. The *x* and *y* axes represent cell numbers. Quadrant D1 represents necrotic cells, quadrant D2 late apoptotic cells, quadrant D3 intact cells, and quadrant D4 early apoptotic cells

The sham-operated rats exhibited very low percentages of apoptotic cells of the CA3 hippocampus cells (Fig. 2). By contrast, experimental DBI led to a significant increase in cell apoptosis. Specifically, while there were only 2.92 ± 0.88 % of apoptotic cells present after 6 h after experimental DBI, the apoptosis rates gradually increased and reached the peak level of 22.26 ± 2.97 % at 72 h after experimental DBI (Fig. 3; Table 1; *p* < 0.001 vs. sham-operated rats).

**Table 1:** Percent of apoptotic cells, and expression of Bcl-2, Bax, and caspase-3 in the rat CA3 hippocampus cells after diffuse brain injury

	Sham-operated animals	Animals exposed to diffuse brain injury					
		6 h	12 h	24 h	48 h	72 h	7 days
Apoptosis rate (%)	1.41 ± 0.64	2.92 ± 0.88*	8.68 ± 1.81*	14.42 ± 1.75*	16.14 ± 1.79*	22.26 ± 2.97*	13.68 ± 3.06*
Bcl-2	2.60 ± 0.89	3.40 ± 0.55*	2.60 ± 0.54	2.20 ± 0.45*	1.60 ± 0.54*	1.20 ± 0.45*	1.80 ± 0.84*
Bax	1.40 ± 0.55	1.80 ± 0.45*	2.20 ± 0.44*	2.40 ± 0.55*	3.00 ± 0.71*	2.60 ± 0.55*	2.00 ± 0.71*
Caspase-3	0	0	1.20 ± 0.45*	2.20 ± 0.45*	2.40 ± 0.55*	3.12 ± 0.24*	2.20 ± 0.44*
Bcl-2/Bax ratio	1.86 ± 1.62	1.89 ± 1.22	1.18 ± 1.21	0.92 ± 0.82	0.53 ± 0.76	0.46 ± 0.82	0.9 ± 1.18

Data are expressed as mean ± SD

* *p* < 0.05 versus sham-operated animals

### Expression of Bcl-2, Bax, and Caspase-3 in the CA3 Hippocampus Cells After Experimental DBI

In sham-treated animals, expression of caspase-3 and Bax was very low, while the expression of Bcl-2 high (Table 1). This pattern of expression of Bcl-2, Bax, and caspase-3 proteins changed in cells of the CA3 region of rat hippocampus after experimental DBI (Table 1). Specifically, expression of Bax peaked at 48 h after brain injury, while the expression of caspase-3 increased to the peak level at 72 h after brain injury (Table 1). However, the expression of Bcl-2 decreased starting from 12 h after brain injury (Table 1). Expressions of these proteins were significant different between sham-operated rats and rats after DBI (*p* < 0.01).

### Relationship Between Percentages of Apoptotic cells, Bcl-2/Bax Ratio, and Expression of Caspase-3

**Fig. 4:**
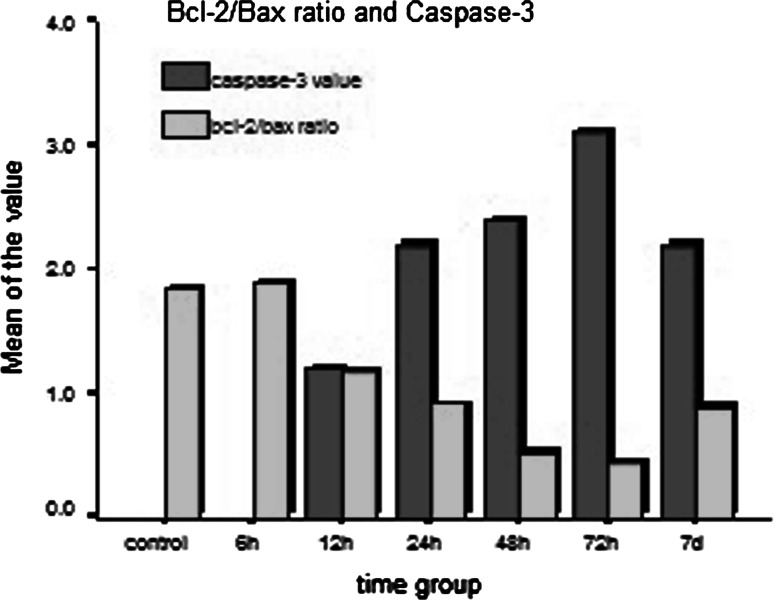
Relationship between the ratio of Bcl-2/Bax expressions and the expression of caspase-3 after DBI

Expression of caspase-3 negatively correlated with the Bcl-2/Bax expression ratio (*r* = −0.982; Fig. 4). The percentage of apoptotic cells reached the peak level at the same time as the expression of caspase-3. However, changes of expression of Bcl-2 and Bax preceded the peak levels of caspase-3 and cell apoptosis.

## Discussion

Cell apoptosis is documented to occur after TBI in humans (1), and the occurrence and extent of neuronal apoptosis are important factors contributing to secondary brain damage after TBI (2). Still, the relationships between expressions of Bcl-2, Bax, and caspase-3 after TBI are still relatively poorly understood. Flow cytometry is a powerful technique for rapid and quantitative analysis of apoptosis with high sensitivity and specificity. The annexin V/propidium iodide double staining method can effectively distinguish between normal, early, and late apoptotic, and necrotic cells [4]. In our study, this staining technique was used for quantitative evaluation of cell apoptosis in the CA3 region of rat hippocampus after DBI. Consistent with previous studies [4, 5], our results demonstrate apoptosis occurring in the CA3 hippocampus in rats after experimental DBI.

The members of Bcl-2 and caspase gene family are important regulators controlling development of cell apoptosis. Bcl-2 inhibits apoptosis and plays an important role in neuroprotection after TBI. By contrast, Bax promotes cell apoptosis, and the ratio Bcl-2 and Bax expression determines the extent of apoptosis [6]. Caspase-3 is an important member of caspase gene family and is directly involved in the morphological and biological changes in apoptotic cells. A previous study [7] performed an in-depth investigation of the role of caspase-3 in cell apoptosis in the TBI model. It was demonstrated that caspase-3 is expressed and activated after TBI. Specifically, caspase-3 proteolysis leads to cell apoptosis, while inhibition of caspase-3 partially protects brain from further injury [4, 8]. However, interactions between the members of Bcl-2 and caspase gene family, and their role in the regulation of apoptosis after TBI is not completely clear. Clark et al. [5] documented expressions of Bcl-2, Bax, and caspase-3 as well as occurrence of apoptosis in human brains after acute injury. However, these authors did not study in more depth the roles of these proteins in apoptosis after brain trauma, as they were limited by the small sample size. Here, we studied expressions of Bcl-2, Bax, and caspase-3 in the CA3 hippocampus of rat brain at different time points after DBI using immunohistochemistry techniques. Along with quantitative analysis of apoptosis, interactions of these proteins in regulation of apoptosis after DBI were also examined. Our results show that expression of Bcl-2 decreased while expression of Bax and caspase-3 gradually increased after brain injury. The percent of apoptotic cells peaked at the same time as expression of caspase-3 peaked. Further, changes in Bcl-2 and Bax expressions occurred prior the peak of both caspase-3 and percent of apoptotic cells, suggesting that Bcl-2, Bax, and caspase-3 are involved in the regulation of cell apoptosis after DBI, with Bcl-2 and Bax being activated during earlier events of the apoptotic pathway. By contrast, caspase-3 is activated by downstream apoptotic signaling. Finally, the change of the Bcl-2/Bax expression ratio preceded the activation of caspase-3. Therefore, we conclude that simultaneous blockage of different regulators of apoptosis may be more efficient in the treatment of secondary brain injury due to neuronal apoptosis after TBI.

## References

[1] Ng I, Yeo TT, Tang WY, Soong R, Ng PY, Smith DR (2000). Apoptosis occurs after cerebral contusions in humans. Neurosurgery.

[2] Marmarou A, Foda MA, van den Brink W, Campbell J, Kita H, Demetriadou K (1994). A new model of diffuse brain injury in rats. Part I: Pathophysiology and biomechanics. Journal of Neurosurgery.

[3] Lenzlinger PM, Marx A, Trentz O, Kossmann T, Morganti-Kossmann MC (2002). Prolonged intrathecal release of soluble Fas following severe traumatic brain injury in humans. Journal of Neuroimmunology.

[4] Zheng JN, Xie SHL, Chen JC (1999). Chin J Immunol.

[5] Clark RS, Kochanek PM, Chen M, Watkins SC, Marion DW, Chen J, Hamilton RL, Loeffert JE, Graham SH (1999). Increases in Bcl-2 and cleavage of caspase-1 and caspase-3 in human brain after head injury. FASEB Journal.

[6] Clark RS, Chen J, Watkins SC, Kochanek PM, Chen M, Stetler RA, Loeffert JE, Graham SH (1997). Apoptosis-suppressor gene bcl-2 expression after traumatic brain injury in rats. Journal of Neuroscience.

[7] Clark RS, Kochanek PM, Watkins SC, Chen M, Dixon CE, Seidberg NA, Melick J, Loeffert JE, Nathaniel PD, Jin KL, Graham SH (2000). Caspase-3 mediated neuronal death after traumatic brain injury in rats. Journal of Neurochemistry.

[8] Barth M, Schilling L, Schmiedek P (2000). Time course of apoptotic cell death after experimental neurotrauma. Acta Neurochirurgica. Supplement.

